# Confirmatory validation of a brief patient-reported outcome measure assessing psychological distress in caregivers of malignant mesothelioma patients: the Mesothelioma Psychological Distress Tool–Caregivers

**DOI:** 10.3389/fpsyg.2024.1444960

**Published:** 2024-10-23

**Authors:** Antonella Granieri, Isabella Giulia Franzoi, Maria Domenica Sauta, Alessandro Marinaccio, Carolina Mensi, Sabrina Rugarli, Enrica Migliore, Ilaria Cozzi, Domenica Cavone, Luigi Vimercati, Federica Grosso, Marinella Bertolotti, Giulia Raimondi, Marco Innamorati, Michela Bonafede

**Affiliations:** ^1^Department of Psychology, University of Turin, Turin, Italy; ^2^Occupational and Environmental Medicine, Epidemiology and Hygiene Department, Italian Workers’ Compensation Authority (INAIL), Rome, Italy; ^3^COR Lombardy, Occupational Health Unit, Fondazione IRCCS Ca’ Granda, Ospedale Maggiore Policlinico of Milan, Milan, Italy; ^4^COR Piedmont, Unit of Cancer Epidemiology, AOU Città della Salute e della Scienza di Torino, CPO Piedmont and University of Turin, Turin, Italy; ^5^COR Lazio, Department of Epidemiology, Lazio Regional Health Service, Local Health Unit 1, Rome, Italy; ^6^Section of Occupational Medicine “B. Ramazzini”, Department of Interdisciplinary Medicine, Regional Operating Center of Puglia (COR Puglia), University of Bari, Bari, Italy; ^7^Mesothelioma and Rare Cancers Unit, Azienda Ospedaliera SS Antonio e Biagio e Cesare Arrigo, Alessandria, Italy; ^8^Research Training Innovation Infrastructure, Research and Innovation Department (DAIRI), AO SS Antonio e Biagio e Cesare Arrigo, Alessandria, Italy; ^9^Clinical Epidemiology Unit, Istituto Dermopatico dell’Immacolata, IDI-IRCCS, Rome, Italy; ^10^Department of Human Sciences, European University of Rome, Rome, Italy

**Keywords:** cancer, oncology, psycho-oncology, burden, caregiver, mesothelioma, occupational cancer, patient-reported outcome measures

## Abstract

**Objective:**

The diagnosis of malignant mesothelioma (MM) can be devastating for both patients and caregivers, who may experience intense suffering from a physical, psychological, and interpersonal perspective. Despite the extensive literature on caregiver distress, there is a lack of validated measures to provide evidence of psychological distress of caregivers of MM patients. Therefore, in a previous study we developed the Mesothelioma Psychological Distress Tool–Caregivers (MPDT-C) to evaluate the specific profile of psychological distress in this population. This paper describes the item selection, factor analysis, and psychometric evaluation of the revised MPDT-C.

**Methods:**

The analyses of this work first aimed to confirm the previous factorial structure. In the case of nonfit, it aimed to find an alternative structure and causes of nonfit in the model. Examination of the fit of the factorial model was conducted using a Bayesian approach.

**Results:**

The final version of the MPDT-C is a 7-item self-report questionnaire consisting of one factor (Burden for the caregiver).

**Conclusion:**

Having a short self-report questionnaire to assess the psychological distress experienced by caregivers of MM patients has several advantages. First, it is suited to epidemiological studies where multiple variables and measures are involved. Second, it is easy to administer and does not take much time to complete. Therefore, the MPDT-C can also be administered in clinical contexts, perhaps when MM patients attend follow-up medical evaluation. Lastly, short measures are less affected by cognitive fatigue, which is very common in a long battery of tests and could affect response reliability.

## 1 Background

Malignant mesothelioma (MM) is a rare, aggressive cancer related to asbestos exposure (Warby et al., 2019), and the progression of the disease is often associated with patient pain, suffering, and uncertainty (Mercadante et al., 2016).

Globally, there is a growing number of MM diagnoses. In developing countries where uncontrolled use of asbestos continues, it is estimated that the number of diagnoses will peak in the next few years due to the MM latency period of 20–45 years after exposure (Carbone et al., 2019). In Italy, the country’s manufacturing activities involved the production and use of asbestos for much of the twentieth century until the use of asbestos fibers was banned in 1992. Thus, the maximum diagnostic peak is expected to be between 2020 and 2030 (Gariazzo et al., 2023; Petrelli et al., 2018; Stella et al., 2023). MM cases and modalities of asbestos exposure are actively investigated by the Italian National Mesothelioma Registry (Registro Nazionale Mesoteliomi—ReNaM), an epidemiological surveillance system based on Operative Regional Centers (Centri Operativi Regionali—COR) (Marinaccio et al., 2018). The seventh report of Registro Nazionale dei mesoteliomi (ReNaM) (2021) estimated that there were 31,572 MM diagnoses from 1993 until the end of 2018 with an average age at diagnosis of 70 years old (DS = 10.6). The Sixth Epidemiological Study of Residents in National Priority Contaminated Sites Report (SENTIERI, 2023) points out that overall mortality from mesotheliomas is three times higher at sites with asbestos, with mortality from pleural MM more than two times higher in the sites with asbestos and port areas.

The diagnosis of MM is devastating due to both the physical symptoms (e.g., loss of appetite, sleep disturbances, pain, and respiratory difficulties) and psychological suffering (e.g., anxiety, depression, feelings of helplessness, social withdrawal, loss of a sense of belonging, and diminished social cohesion) (Bonafede et al., 2020; Dooley et al., 2010; Granieri et al., 2013; Guglielmucci et al., 2018; McCormack et al., 2012; Mesothelioma UK, 2012; Moore et al., 2008; Northouse et al., 2012).

Previous research has shown that cancer diagnosis, the disease experience, and caregiving-related tasks can have a significant impact on caregivers, who may experience intense suffering from a physical, psychological, and interpersonal perspective (Bedaso et al., 2022; Teixeira et al., 2018; Lee Wong et al., 2020).

Not only is there a drastic change in daily activities, work, and relationships, but a process of reformulation of family roles often also begins. Following diagnosis, caregivers can experience a conflict between different social roles. They also have to cope with their loved one’s need for support, both emotionally and at the level of managing various visits, including communication with health care personnel (Kim and Given, 2008). Often caregivers may state a general need for help, but rarely get to define their specific needs (Sandén et al., 2019). This can lead to intense feelings of ineffectiveness and impotence (Bonafede et al., 2022), as well as somatopsychic dysregulation, which can lead to affective dysregulation, difficulties in symbolization and mentalization, and dysregulated psychophysiological functioning in immune and inflammatory terms (Bijoor et al., 2016; Coumbe and Groarke, 2018; Kim et al., 2022; Renna et al., 2020).

Feelings such as helplessness and fear of losing their loved one can worsen their experience (Almeida et al., 2019; Granieri et al., 2018; Guglielmucci et al., 2018) and can lead to anticipatory grief (Li et al., 2022). Moreover, when facing the death of a loved one, caregivers can experience a prolonged period of mourning, a psychic state characterized by a frozen internal emotional state and a disinvestment of libido in the external world (Borgogno et al., 2015; Kustanti et al., 2023).

Previous studies have pointed out the importance of assessing the psychological needs of caregivers of patients with MM in order to support them throughout the care and end of life of their loved ones. In addition, assessment of caregivers’ needs would help provide psychological interventions to meet their caregiving needs (Ejegi-Memeh et al., 2022; Granieri, 2015, 2016; Guglielmucci et al., 2014; Moore et al., 2008).

However, despite the extensive literature on caregiver distress, there is a lack of validated Patient-Reported Outcome Measures (PROMs) to provide evidence of the psychological distress of caregivers of MM patients. Thus, we aimed to develop a brief PROM (the Mesothelioma Psychological Distress Tool–Caregivers, MPDT-C) to evaluate the specific profile of psychological suffering in this population (Bonafede et al., 2022). Its preliminary validation resulted in a 45-item questionnaire with a three-factor structure: Secondary Traumatic Stress, Engagement in Caring, and Meaningful Cognitive Restructuring.

However, we wanted to provide a revised, shorter version of the MPDT-C, easier to administer, less time-consuming and easy to combine with other questionnaires. Thus, this paper describes the item selection, factor analysis, and psychometric evaluation of the revised MPDT-C. The analyses of this work first aimed to confirm the factorial structure found in the work of Bonafede et al. (2022). As a secondary objective in the case of nonfit, the present study aimed to find an alternative structure and causes of nonfit in the model. A Bayesian approach was used to examine the fit of the factorial model.

## 2 Materials and methods

### 2.1 Participants and procedures

Data were collected through a prospective observational multicentric study conducted in clinical settings. Participants were recruited through the Apulia, Lombardy, Lazio and Piedmont CORs of the ReNaM, the SS Antonio e Biagio e Cesare Arrigo Hospital of Alessandria (Piedmont), and the Santo Spirito Hospital of Casale Monferrato (Piedmont). We enrolled caregivers of patients diagnosed with definite, probable or possible malignant mesothelioma according to the Italian National Mesothelioma Registry (ReNaM), regardless of location and stage.

At the SS Antonio e Biagio and Cesare Arrigo Hospital of Alessandria and the Santo Spirito Hospital in Casale Monferrato, subjects were contacted and enrolled by in-person interview during hospital follow-up visits and written informed consent to participate in the study was obtained. At the CORs, which are not involved in patient care, subjects were contacted and enrolled during routine activities. When caregiver access at the CORs was possible, written informed consent was collected, while when due to the protracted nature of the COVID-19 pandemic in-person access was not feasible, informed consent was collected by telephone with a written statement. Informed consent was obtained from participants either by in-person collection, verbally in the case of telephone calls, or through a combination of verbal explanation and on-screen projection using videoconferencing tools. Due to the COVID-19 pandemic, administration of the test battery was done in person, via videoconferencing tools, or via the telephone. In-person administration was always preferred, where feasible.

### 2.2 Measures

Participants were administered the preliminary version of the MPDT-C (47 items) (Bonafede et al., 2022), as well as a specific battery of tests designed to assess construct validity:

- Secondary Traumatic Stress Scale-Italian Version (STSS-I) to assess caregivers’ post-traumatic experiences and secondary traumatization (Setti and Argentero, 2012). The STSS consists of 15 items, which are rated on a 5-point Likert scale from 1 (never) to 5 (very often). The scale includes two subscales: intrusion (STSS-IN) and arousal (STSS-AR).
- Caregiver Burden Inventory (CBI) to assess the burden related to the role of caregiver (Novak and Guest, 1989; Marvardi et al., 2005). The CBI consists of 24 items, which are rated on a 5-point Likert scale from 0 (not at all disruptive) to 4 (very disruptive). It comprises five subscales: Time-related distress (CBI-T/dep-B), Developmental distress (CBI-Dev-B), Physical distress (CBI-Phys-B), Social distress (CBI-Soc-B) and Emotional distress (CBI-Emo-B). A total score is also calculated (CBI-TOT).
- Personal Need for Structure Scale (PNS) to assess the tendency to engage in cognitive activities (Neuberg and Newsom, 1993). The PNS consists of 12 items, that are rated on a 6-point Likert scale from 1 (strongly disagree) to 6 (strongly agree). It comprises two subscales: Desire for Structure (PNS-DFS) and Response to Lack of Structure (PNS-RLS).

### 2.3 Statistical analyses

All analyses were performed with JASP 0.17.3.0, and MPlus version 7 (Muthén and Muthén, 1998–2010) and Bayesian confirmatory factor analysis were used to investigate the dimensionality of the scale. Items that specifically assessed the burden experienced by the caregivers in relation to their family members were selected.

First, an exploratory factor analysis was conducted. Bartlett’s test of sphericity and the Kaiser–Meyer–Olkin (KMO) test were performed to assess whether the data were suitable for factor analysis, and a specific Measure of Sampling Adequacy (MSA) was considered for each item. Only items with a high MSA and with high factor loadings (≥0.40) were selected through an iterative process. Moreover, a simple structure pattern was looked for, where all items significantly and strongly loaded (≥0.40) on their respective factors and had low loadings (<0.40) on the other factors.

As a final step, the model retrieved in the previous exploratory factor analysis was evaluated by means of a Bayesian confirmatory factor analysis with a Markov chain Monte Carlo (MCMC) algorithm. We used the GIBBS sampling algorithm and 100,000 post burn-in iterations (Taylor, 2019). Weak informative priors [N(0, 1.0)] were used for the hypothesized factor loadings. The sensitivity of the model to priors was inspected, comparing the hypothesized model with two competing models which increasingly favor the null hypothesis for factor loadings [N(0, 0.25) and N(0, 0.10)]. Furthermore, a final model which included free cross-loadings with a low-variance normal prior [N(0, 0.01)] was assessed (Taylor, 2019). The model fit was evaluated using the Bayesian Posterior Predictive Checking (PPC) and the Posterior Predictive *p*-value (PPP; Muthén and Asparouhov, 2012). The fit of the model was based on the PPC confidence interval crossing the zero and PPP > 0.05.

Internal consistency is reported as Cronbach’s *α* and McDonald’s *ω* with 95% credible intervals (Pfadt et al., 2023). As a measure of convergent validity, we report Pearson correlation coefficients between the MPDT-C, the STSS-17, CBI-24, and PNSS-12. Statistical significance of correlations indices is reported as the number of times (BF_10_) the support for the alternative hypothesis (i.e., the measures are associated) is larger than that for the null hypothesis (i.e., the measures are not associated). For example, BF_10_ > 10 means that the support for the alternative hypothesis (i.e., the measures are associated) is more than 10 times larger than that for the null hypothesis.

Finally, cutoff scores for the MPDT-C were calculated by using quartiles. Low MPDT-C scores were considered within the 1st quartile (≤25th percentile); moderate MPDT-C scores between the 1st quartile and the 3rd quartile (>25th percentile and <75th percentile); and high MPDT-C score above the 3rd quartile (≥75th percentile).

## 3 Results

A total of 178 caregivers (75.56% female) with a mean age of 56.70 (SD = 12.31; range = 42–83) were recruited: 108 at the Lombardy COR (60.67%), 39 at the Apulia COR (21.91%), 12 at the SS Antonio e Biagio e Cesare Arrigo Hospital of Alessandria (6.74%), 10 at the Santo Spirito Hospital of Casale Monferrato (5.61%), 6 at the Piedmont COR (3.37%) and 3 at the Lazio COR (1.68%).

### 3.1 Exploratory factor analysis

The three-factor solution found in the original study (Bonafede et al., 2022) was not confirmed by the Bayesian confirmatory factor analysis (Bayesian posterior predictive checking using 95% confidence interval for the difference between the observed and the replicated Chi-square values = 589.675/889.154; posterior predictive *p*-value <0.001). Moreover, when inspecting the model, several items reported low factor loadings, suggesting the presence of problematic items and that the model should be revised.

Twenty-one items were selected on the basis of their meaning, and exploratory factor analysis was conducted (see Table 1 for initial KMO and MSA values).

**Table 1:** Initial KMO measures.

KMO measure of sampling adequacy	
	MSA
Overall	0.713
Item 2	0.780
Item 4	0.480
Item 6	0.769
Item 7	0.676
Item 8	0.700
Item 11	0.757
Item 14	0.680
Item 17	0.678
Item 20	0.728
Item 21	0.771
Item 24	0.731
Item 27	0.523
Item 28	0.718
Item 36	0.604
Item 37	0.754
Item 40	0.800
Item 41	0.742
Item 42	0.646
Item 44	0.781
Item 45	0.724
Item 47	0.617

The iterative process led to the inclusion of seven items, which all significantly loaded on a single dimension (see Table 2).

**Table 2:** Factor loadings and unique variances of the item selected.

	Factor loadings	Uniqueness
Item 6	0.624	0.610
Item 20	0.472	0.777
Item 21	0.667	0.556
Item 37	0.518	0.731
Item 40	0.845	0.286
Item 41	0.443	0.804
Item 42	0.438	0.808

The “principal axis factoring” extraction method was used in combination with a “oblimin” rotation.

### 3.2 Bayesian confirmatory factor analysis

We performed a Bayesian confirmatory factor analysis, testing the fit of the MPDT-C 7-item model. The model fit the data (Bayesian posterior predictive checking using 95% confidence interval for the difference between the observed and the replicated Chi-square values = −17.47/31.24; posterior predictive *p*-value = 0.28), and all items loaded significantly (<0.001) and >0.40 on the single latent dimension (between 0.408 and 0.703) (see Table 3).

**Table 3:** Factor loadings of the 7-item version of the MPDT-C according to Bayesian confirmatory factor analysis.

	Factor loadings	95% CI – lower bound	95% CI – upper bound
Item 6	0.544	0.380	0.679
Item 20	0.555	0.392	0.689
Item 21	0.691	0.535	0.810
Item 37	0.662	0.504	0.786
Item 40	0.703	0.560	0.816
Item 41	0.408	0.236	0.559
Item 42	0.674	0.496	0.809

95% CI, 95% credibility intervals.

Sensitivity analysis to priors did not suggest an effect of priors on parameter estimates. When using informative priors which increasingly favor the null hypothesis for factor loadings [N(0, 0.25) and N(0, 0.10)], the models indicated the same adequate fit (moderate informative prior: Bayesian posterior predictive checking using 95% confidence interval for the difference between the observed and the replicated Chi-square values = −17.63/31.04, posterior predictive *p*-value = 0.27; strong informative prior: −17.66/31.16, posterior predictive *p*-value <0.27). Changes in estimated factor loadings were all below 10% with moderately informative priors, and <20% with strongly informative priors.

### 3.3 Psychometric properties of the MPDT-C

The latent dimension had a McDonald’s *ω* posterior mean of 0.70 (95%CI = 0.62/0.76, posterior probability 0.70 < *ω* < 0.90 = 0.51; Cronbach *α* = 0.70, 95%CI = 0.63/0.76, posterior probability 0.70 < *α* < 0.90 = 0.59). Thus, internal consistency estimates were satisfactory, but the posterior probabilities for the reliability indices to be included in the 0.70/0.90 range were below 70%.

Table 4 presents the reported correlation indices (Pearson’s *r*) between the MPDT-C and concurrent measures. The MPDT-C significantly correlated with all dimensions of the STSS, all dimensions of the CBI, and the PNS-RLS except for the PNS-DFS (*r* = 0.03).

**Table 4:** Bayesian correlation coefficients for the 7-item MPDT-C and other constructs.

	MPDT-C	Lower 95% CI	Upper 95% CI
STSS-AR	0.44***	0.315	0.552
STSS-IN	0.34***	0.204	0.464
CBI-TOT	0.48***	0.362	0.588
CBI-Dev-B	0.54***	0.433	0.641
CBI-Phys-B	0.42***	0.298	0.539
CBI-Soc-B	0.36***	0.230	0.485
CBI-Emo-B	0.29***	0.156	0.424
CBI-T/dep-B	0.18	0.038	0.321
PNS-DFS	0.03	−0.112	0.180
PNS-RLS	0.26**	0.125	0.398

***BF_10_ > 100; **BF_10_ > 30, *BF_10_ > 10; CI, credibility intervals.

### 3.4 Scoring and cutoff scores

The final version of the MPDT-C is a 7-item self-report questionnaire consisting of one factor assessing the burden experienced by the caregiver via a 4-point Likert scale (1 = “completely/totally disagree” to 4 = “completely/totally agree”). Scores are calculated by giving each item the value that the subject selected on the corresponding Likert scale. The total score is the sum value of the scores obtained in the seven items. The mean score was 13.91 (SD = 3.20; range 7–22). MPDT-C scores of 12 or less indicate a low burden for the caregiver; MPDT-C scores greater than 12 and less than 16 indicate a moderate burden for the caregiver; MPDT-C scores of 16 or greater indicate a high burden for the caregiver.

## 4 Discussion

In this paper we have presented the item selection, factor analysis, and psychometric evaluation of the revised MPDT-C (Bonafede et al., 2022).

First, only items that specifically assessed the burden experienced by the caregivers when taking care of their family members were selected (Appendix 1). The decision to only include these items allowed us not only to exclusively focus on the personal experience of caregivers of MM patients, but also to provide a more streamlined tool that is more focused on the distress experienced by caregivers.

Our results indicated that a unidimensional structure, with seven items, fitted the data best. Moreover, the posterior predictive *p*-value did not change with priors favoring the null hypothesis, indicating the strong stability of the 7-item model of the MPDT-C. The MPDT-C yielded satisfactory internal consistency and construct validity (i.e., associations with other variables) and shared a strong association with the Arousal subscale of the STSS and a moderate association with the Intrusion subscale.

These results are in agreement with previous research reporting post-traumatic symptoms in caregivers, not only in relation to MM (Bonafede et al., 2020; Sherborne et al., 2022), but also in relation to other terminal illnesses (Alfheim et al., 2019; Jia et al., 2015; Wang et al., 2023). Cancer deeply challenges individuals’ sense of identity: they are confronted with feelings of helplessness, vulnerability, separation, and death (Granieri, 2016). Psychologically, it is often experienced as a catastrophe, not only by those who experience it physically in their bodies but also for those who care for them. Caregivers play an essential role in supporting their loved ones through this journey, taking on a wide range of responsibilities, such as helping with daily activities and providing emotional support. As the illness progresses, caregivers often witness the physical and emotional deterioration of their loved ones, which can have a profound impact on their mental health. Often their energies are focused on caring for the family member and they are burdened with the responsibility to “think for the patient as well” while losing sight of their own needs (Breen et al., 2022).

Caregivers’ lives change alongside those of the patients. In a daily routine punctuated by suffering, responsibilities, and time and energy devoted to caregiving, violent emotions can surface in the caregiver’s mind. These emotions can be difficult to express and are not always conscious, fueling feelings of shame and guilt. Touching on the desire to reclaim time for themselves, to get away from their loved one, or even to feel somehow “liberated” by the latter’s death, such feelings can be an intolerable emotional experience. Faced with the loss of one’s family member, the caregiver may sink into a “nameless terror” (Bion, 1962) where affects are perceived and experienced only at the bodily level and it is impossible for the subject to symbolize and integrate them into a coherent discourse (Caspi, 2018; Segal, 2019).

The MPDT-C also shared a moderate and strong association with all the Caregiver Burden Inventory dimensions, except for the Time-dependent subscale. These results highlight the extreme level of burden experienced by caregivers (Girgis et al., 2019), which is associated with several psychopathological symptoms, such as anxiety and stress. Caregivers may experience compassion fatigue (Lee et al., 2009), which can be defined as feeling emotionally depleted from the constant caregiving demands.

The unthinkability and unlivable nature of grief are linked on the one hand to the severity of the loss, which seems unbearable, and on the other hand to the fragility of the structure of the self, which lacks a psychic skin and mental perceptual containment to allow this experience to be held together. The nature of the prognosis that is often so inauspicious and the low life expectancy often make the caregiver experience emotions such as aggression and anger over an unfair fate and future. These emotions seem to occupy their entire internal world, hindering their ability to connect with their vulnerable, helpless, and needy inner parts and the initiation of the grieving process (Granieri, 2017).

Finally, the MPDT-C significantly correlated only to the Lack of Structure factor of the Personal Need for Structure scale. This factor refers to the extent to which individuals respond to non-structured and unpredictable situations (Neuberg and Newsom, 1993). Individuals who do not like uncertainty and/or changes of plans at the last moment score higher in this scale. These findings could reflect the uncertainty associated with the progression of MM, which forces caregivers to devote all their energy and attention to their loved ones, preventing them from having a more structured life including other activities and personal time (Ejegi-Memeh et al., 2022). The fear of themselves getting cancer and leaving their loved one without help, while present, frequently remains unexpressed and undermines their sense of security (Sandén et al., 2019). This emotional turmoil, difficult and painful to think about, is very common in the inner worlds of cancer patients and their caregivers; it can end up inhibiting their action and freezing their emotional and relational lives (Kleine et al., 2019; O’Toole et al., 2017).

### 4.1 Clinical implications

Having a shorter self-report questionnaire to assess the psychological distress experienced by caregivers of MM patients has several advantages. First, it is suited for epidemiological studies where multiple variables and measures are involved. Second, short scales are easy to administer and do not take up much time. Therefore, this could also allow the administration of the MPDT-C to caregivers in clinical contexts, perhaps when MM patients go to follow-up medical evaluation. Lastly, short measures are less affected by cognitive fatigue, which is very common in a long battery of tests and could affect response reliability (Ackerman and Kanfer, 2009).

### 4.2 Study limitations

Although our study produced interesting results, it has some limitations. For example, self-report measures may be influenced by response biases such as social desirability (Van de Mortel, 2008). In addition, respondents may not be able to assess themselves accurately; the wording of the questions may be confusing or mean different things to different people; the questions are all subject to biases introduced by previous responses, whether they relate to recent or important experiences, and other factors. Moreover, we did not examine the stability of the factor structure of the MPDT-C at the item level, nor did we assess its discriminant validity. Finally, while Bayesian factor analysis is the recommended approach when dealing with small samples, a larger sample with a more balanced number of women and men would help in generalizing the results to the broader population.

### 4.3 Conclusion

In the area of mental health promotion and protection, it is important to identify factors related to diagnosis, prognosis, and treatment for individuals and families affected by the experience of oncologic disease, including grief and loss.

The use of the MPDT-C may be of great assistance in identifying caregivers who are experiencing greater distress and grief and who may benefit most from the activation of psychological and psychotherapeutic interventions, both during the course of the illness and after the death of loved ones. If the literature on possible psychological interventions for patients with malignant mesothelioma is sparse, there are even fewer articles that have explored possible interventions for caregivers (Franzoi et al., 2024). One of the interventions proposed to caregivers of mesothelioma patients is the Brief Psychoanalytic Group (BPG), that was developed and implemented at the Contaminated Site of Casale Monferrato (Granieri et al., 2018). The BPG model comprises 12 sessions aimed at patients and caregivers immediately after diagnosis and involves working on a somatopsychic focus, a common and recurring theme within the group that simultaneously relates to the physical symptoms associated with the illness and to the emotions, affects and fantasies associated with it. Therapeutic work on the somatopsychic focus enables the group to integrate the intense emotions associated with death and grief and to find healthier ways of interacting on an intrapsychic and interpersonal level. A second level of intervention, developed specifically for Contaminated Sites, also provides for the activation of a multifamily group for the entire community (Granieri, 2016, 2017), an “open door” group aimed at patients, family members, healthcare professionals and, in general, anyone who wishes to participate, in order to offer the entire population a psychological space and the opportunity to work simultaneously on the individual, family and social dimensions of the mind.

The opportunity to express emotions and thoughts in a professionally equipped setting can provide valuable opportunities for caregivers to acknowledge the efforts they are making in caring for their loved one, but also to recognize their personal needs. When caregiving is appropriately supported from a psychological perspective, it is an experience that can open up learning opportunities: the caregiver may feel that their sense of self-efficacy is strengthened, and they may discover previously untapped resources for coping with the crisis.

## Data Availability

The raw data supporting the conclusions of this article will be made available by the authors, without undue reservation.
