# Supplementary material for: Confirmatory validation of a brief patient-reported outcome measure assessing psychological distress in caregivers of malignant mesothelioma patients: the Mesothelioma Psychological Distress Tool–Caregivers
Source: Front Psychol. 2024 Oct 23;15:1444960. doi: 10.3389/fpsyg.2024.1444960 (PMC11541108; doi:10.3389/fpsyg.2024.1444960)
Supplement: Supplementary file 1 [file Table_1.DOCX]

**Appendix 1.**

**MESOTHELIOMA PSYCHOLOGICAL DISTRESS TOOL – CAREGIVERS (MPDT-C)**

Completion date Interviewer_____________________________________

Dear family member, please indicate your degree of agreement/disagreement with the statements below.

1. **Totally disagree**
2. **Disagree**
3. **Agree**
4. **Totally agree**

After your family member’s diagnosis

| 1. 6 | I often avoid thinking about the things that I can no longer do on a daily basis because of my family member’s illness/I’ve often avoided thinking about the things that I could no longer do on a daily basis because of my family member’s illness. | 1 | 2 | 3 | 4 |
| --- | --- | --- | --- | --- | --- |
| 1. 20 | I feel/felt very lonely in my role as a caregiver. | 1 | 2 | 3 | 4 |
| 1. 21 | I have had relationship problems because of my role as a caregiver to the sick person. | 1 | 2 | 3 | 4 |
| 1. 37 | My family member’s illness has changed the way others look at me. | 1 | 2 | 3 | 4 |
| 1. 40 | I feel/felt caged at this time of life/during caregiving. | 1 | 2 | 3 | 4 |
| 1. 41 | I try/tried to do as many things as possible during the day to take my mind off thinking about my family member’s illness. | 1 | 2 | 3 | 4 |
| 1. 42 | My role as a caregiver to the sick person causes/caused me embarrassment. | 1 | 2 | 3 | 4 |

Administration time:
